# Response of soybean Cd to soil Cd and pH and its associated health risk in a high geological background area in Guizhou Province, Southwest China

**DOI:** 10.1371/journal.pone.0312301

**Published:** 2024-10-22

**Authors:** Xiaosong Tian, Guanqun Chai, Lina Zhu, Junlin Zhou, Qing Xie, Kangwen Zhu

**Affiliations:** 1 College of Resources, Environment and Safety, Chongqing Vocational Institute of Engineering, Chongqing, China; 2 Institute of Soil and Fertilizer, Guizhou Academy of Agricultural Sciences, Guiyang, China; 3 School of Smart City, Chongqing Jiaotong University, Chongqing, China; Center for Research and Technology Transfer, VIET NAM

## Abstract

This study comprehensively examined the accumulation of cadmium (Cd) in soybeans grown in low- and high-Cd soils around the high geological background areas in Guizhou province. The aim was to analyze the relationship between soybean Cd and soil pH and soil Cd, alongside assessing the potential carcinogenic and non-carcinogenic risks associated with Cd in soybeans. Cd content of soybeans cultivated in the high-Cd area (0.430 mg/kg) was significantly higher than that in low-Cd areas (0.156 mg/kg) (P < 0.05). Biological concentration factors (BCFs) of soybean for Cd in low- and high-Cd areas were 0.282 and 0.314, respectively, with no significant differences (*P* > 0.05). Multiple linear regression results indicated that soil pH was a determining factor for Cd accumulation in soybeans in both areas. Furthermore, soil pH and soil Cd could accurately predict Cd accumulation in soybeans according to the neural network model. These findings suggest that regulating soil pH could reduce Cd accumulation in soybeans in areas with high geological background. In both areas, there was no significant non-carcinogenic risk for the adult population (HQ value < 1) through soybean consumption. However, according to the Monte Carlo model, the percentage of Cd in soybeans exceeding the acceptable range (CR value > 1.00 × 10 ^−04^) in areas was 99.18%, indicating an unacceptable carcinogenic risk for the adult population. Our discussion revealed that reducing the soybean intake and increasing soil pH did not effectively lower the carcinogenic risk of Cd in soybeans to an acceptable range (CR value ≤ 1.00 × 10 ^−04^). These findings necessitate further exploration of alternative remediation strategies to ensure the safe production of soybeans, such as screening for low-Cd accumulation soybean varieties and implementing the combined remediation strategies.

## 1. Introduction

Soybean (*Glycine max*), which originated in China over 5000 years ago and is now widely cultivated worldwide, is an essential agricultural product owing to its high oil and protein content [1, 2]. Soybean is the raw material for soybean oil and soy-based products (such as soymilk and tofu) and is extensively consumed by the Asian population. It is well known that soybeans have become an essential and irreplaceable part of the Chinese diet [2, 3]. Therefore, the safety of soybeans and its toxic element contents in soy-based foods have attracted significant research attention [4–6]. Soybean has been reported to accumulate more poisonous elements than other crops, severely threatening food safety and people’s health [3, 7]. Compared to other toxic elements, Cd is one of the primary contaminants in agricultural lands, with the highest exceeding standard rate in China, according to the communique on soil contamination released by the Ministry of Ecology and Environment of the People’s Republic of China (2014). Furthermore, soil Cd can be readily translocated to soybeans, leading to a content exceeding the food standards (0.2 mg/kg in GB 2762–2017) [8]. In general, the average content of Cd in soybeans (0.32 mg/kg) in southern China is much higher than that in other countries (e.g., Argentina, Germany, and Brazil, which are at 0.150, 0.130, and 0.023 mg/kg, respectively) [7]. In addition, Cd enrichment capacity of soybeans is higher than the other toxic elements (such as Hg, As, Pb, and Cr) [9, 10], suggesting that soybeans are liable to absorb soil Cd and thus harmful to human health via the food chain. With the long-term intake of Cd-contaminated food, Cd will accumulate in the kidneys, causing damage to kidney function. The tolerable intake (acceptable daily intake, ADI) recommended by FAO / WHO is 25 μg per kg of body weight per month (58 μg for a 70 kg person per day) [11].

Generally, the high accumulation of Cd in soybeans is likely ascribed to the increased bioavailability and content of soil Cd, and the enrichment capacity of the soybean variety. The bioavailability of soil Cd depends on soil physicochemical characteristics and Cd-pollution degree [12–14], and the enrichment capacity of soybeans for Cd is mainly influenced by genotypic difference [15–17]. Soybeans can efficiently uptake and accumulate Cd with high bioavailability from agricultural soils. Soil pH is a critical geochemical factor that determines the bioavailability of soil Cd and, therefore, controls the Cd accumulation in soybeans. Previous studies have observed that soybeans can quickly accumulate Cd from acidic or neutral soils, resulting in high-Cd soybean and associated health risks [5, 6, 9, 14], indicating that soil pH is an essential variable to predict the bioavailability of soil Cd and Cd accumulation in crop products [12, 13, 18–20]. Usually, Cd-containing compounds with low solubility in calcareous soils with high pH values limit the Cd bioavailability [21, 22]. Accumulation characteristic of soil Cd is another crucial factor affecting the Cd content of soybean grains. Cd content and fraction in soils are closely related to the sources, such as atmospheric deposition and industrial/agricultural activities [23–25]. The elevated Cd level in farmland due to mining/smelting activities, emission sources, and fertilizer application poses high risks of Cd accumulation in soils and food crops such as soybeans [5, 9]. Cd content in agricultural soils may also decrease during the runoff or soil acidification in the long run due to a high proportion of extractable Cd by weak acid in soils [23, 26]. However, the soil Cd usually shows an increasing trend when the input fluxes of soil Cd from atmospheric dry and wet deposition, fertilizer application, and irrigation water are more significant than the Cd output fluxes [23, 25, 27]. Previous research has discussed that Cd in agricultural soils usually originates from geological processes and anthropogenic activities [27]. Many studies have focused on the influence of anthropogenic activities on soil Cd accumulation and its associated risks [28–30]. However, soil and crop pollution caused by geological anomalies and their induced social issues have often been neglected [31, 32]. There are large areas with high Cd geological background anomalies in south China, such as Guizhou and Guangxi provinces [33, 34]. The background value of soil Cd (0.66 mg/kg) in Guizhou province exceeds the standard limit value for soil environmental quality set by GB15618–2018, presenting a potential health risk for residents while also indicating the low solubility of soil Cd on account of the properties of calcareous soil with a high pH [34–36]. Soil Cd bioavailability in high geological background areas could increase when the soil acidification intensifies, thus increasing the Cd accumulation in crops and ultimately exacerbating the exposure risk to crop Cd [26, 37]. Therefore, understanding the effects of soil Cd and pH on Cd accumulation in soybeans in areas with the high geological background is of great practical significance.

Nevertheless, the response of Cd accumulation in soybeans to soil Cd versus soil pH and health risks of soybean Cd in high Cd geological background areas has not been researched sufficiently. Hence, we selected two regions with a significant variance in soil Cd content in the typical high Cd geological background area in Guizhou province to analyze Cd accumulation in soybeans. The main objectives of this study are as follows: 1) investigate the accumulation characteristics of Cd in soybeans in response to the soil Cd and soil pH; 2) assess the health risks (the carcinogenic and non-carcinogenic risks) from exposure to soybean Cd in the adult population using Monte Carlo simulation; and 3) discuss whether the accumulation of soybean Cd can meet the requirements of agricultural products in the hypothetical scenario. Ultimately, this study is intended to provide a solution with guiding significance for the safe production of soybeans and safe utilization of Cd-contaminated agricultural soils in the area with high Cd geological background.

## 2. Materials and methods

### 2.1. Study areas and sampling

The sampling sites are located in the demonstration field of northern Guizhou province, a high Cd geological background area. This study involved the low- and high-Cd areas in Yangjiawan and Linkou, respectively, in S1 Fig. The low-Cd area ranged from 105°19′35″ E to 105°19′55″ E and from 27°38′40″ N to 27°38′55″ N; the high-Cd area ranged from 104°59′20″ E to 104°59′35″ E and from 27°12′5″ N to 27°12′25″ N. Soil types in both regions are yellow soils, and their parent rocks are carbonate rocks. Both regions are typical agricultural areas without industrial sources associated with heavy metals. In a previous investigation, the soil pH values in the high- and low-Cd areas were 4.13–8.19 and 4.19–7.31, respectively. When the soybeans (*Qian Dou*) in low- and high-Cd areas were ripe, soybean and corresponding soil samples were collected in 2021. Three to five randomly collected soybean subsamples and their related soil subsamples were collected from each field to form the mixed soybean and soil samples, respectively. A total of 36 soybean samples and soil samples were gathered according to the national standard (DZ/T 0295–2016) [38]. Soil samples of approximately 1 kg were collected and placed in sample cloth bags, and soybean samples of about 2 kg were gathered in mesh bags. Soybean samples were returned to the laboratory, cleaned with deionized water, and dried with absorbent paper. Subsequently, the cleaned soybean samples were dried at 105°C for one hour and at 65°C to constant weight in an oven. Soil samples were air-dried at room temperature in the sample air-drying room. The dried soybean samples were ground using a three-dimensional shock ball mill (TJS-325, Techin, China); and air-dried soil samples were ground with an agate mortar and passed through 10-mesh and 100-mesh nylon sieves. The pretreated samples were stored in zipped plastic bags to prevent moisture from entering before further analysis. The sample pretreatment was conducted individually to avoid cross-contamination of the soybean and soil samples. Sample pretreatment methods of soil and soybeans followed procedures from other studies [3, 7].

### 2.2. Determination of Cd concentration in the soil and soybean

Soil Cd content was determined according to the National Environmental Protection Standards of the People’s Republic of China (HJ 1315–2023) [39]. The weighed soils of the < 100 mesh were put into a Teflon digestion tank. Next, the Teflon digestion tanks added 3 mL of concentrated hydrochloric acid (HCl) and 9 mL of concentrated nitric acid (HNO_3_). Their lids were closed, and the tanks were placed in a microwave device. The sample temperature was raised to 180°C for three steps within 17 minutes and maintained at 180°C for 25 minutes. The digestion solution was transferred into the crucible, 2 mL of concentrated hydrofluoric acid (HF) was added and subsequently heated on an electric heating plate at 120°C until the liquid became viscous, and 1 mL of concentrated perchloric acid (HClO_4_) was added and then heated on an electric heating plate at 160°Cuntil the liquid became viscous again. After cooling to room temperature, the Cd was dissolved using 2% nitric acid, and the solution was transferred to a volumetric bottle of 50 mL, with the volume set to 50 mL. The concentration of Cd in the digestion solution was determined using an inductively coupled plasma mass spectrometer (ICP-MS, iCAP-Q, USA). The method detection limit (MDL) of Cd was 0.05 mg/kg. For quality assurance and quality control (QA/QC), the standard reference certified standard material (GBW 07405) was inserted in each digestion group to evaluate the accuracy and precision of the analysis process and method. The certified standard material was from the Center for National Standard Reference Material of China, and the recovery rate of Cd in certified standard material was 92%– 103%. Soil pH was determined with a volume ratio of water to soil of 2.5: 1 (mL/g) using a pH mV/pH detector (PHS-3C, LEICI, China).

The Cd content in soybean was determined by ICP-MS (iCAP-Q, USA) after the soybean samples were digested by the nitric acid-microwave method according to the National Standard of the People’s Republic of China–National Standard for Food Safety (GB 5009.268–2016) [40]. In detail, the weighed soybean samples were placed in the microwave digestion tanks, 5–10 mL of nitric acid was added, and overnight maintenance was performed. The digestion temperature of soybean samples was raised to 190°C within 15 minutes in three steps and maintained at 190°C for 20 minutes in a microwave digestion instrument. After cooling, the digestion tanks were put on the digital display electric heating plate, heated at 100°C for 30 min, and then transferred to a volumetric flask. During the analysis process, the certified standard material (GBW 100348) was used for QA/QC, and the recovery rate of Cd was 98%– 100%. The MDL of Cd was 0.001 mg/kg.

### 2.3. Health risk assessment of soybean Cd

Firstly, this study used correlation and multiple linear regression methods to explore the quantitative relationship between soybean Cd, soil pH, and soil Cd. Secondly, the health risk assessment of soybean Cd for the adult population was discussed by the coupling the health risk assessment model with the Monte Carlo model. Finally, the prediction model of soybean Cd with the neural network model was used to evaluate the health risks of soybean Cd under hypothetical scenarios, as plotted in S2 Fig.

#### 2.3.1. Health risk assessment based on the concentration

Many researchers have used the health risk assessment model developed by the US EPA to evaluate non-carcinogenic and carcinogenic effects on humans. This study assessed the health risks based on soybean consumption. The exposure to Cd in soybean can be described by the average daily dose (ADD, mg/kg/day). The ADD index is computed by Eq (1):

$$ADD_{Cd,ing}=\frac{C_{Cd,c}\times IR_{Cd,ing}}{BW}\times 10^{-3}$$ (1)

Where *C*_*Cd*,*c*_ represents the Cd concentration in the soybean sample (mg/kg); *IR*_*Cd*,*ing*_ represents the daily ingestion rates of soybeans (37 g/day) [10, 41]; and BW is the body weight of the exposed individual (adults, 63 kg). Based on the average daily dose of Cd, the non-carcinogenic risks from the consumption of soybeans were evaluated individually by the hazard quotient (*HQ*_*Cd*_) as shown in Eq (2) [42–44].

$$HQ_{Cd}=\frac{ADD_{Cd,ing}}{RfD_{Cd,ing}}$$ (2)

Where *RfD*_*Cd*,*ing*_ is the reference exposure dose of Cd (1.0 × 10−^03^ mg/(kg·day)) via soybean ingestion [45, 46]. *HQ*_*Cd*_ < 1 indicates that the non-carcinogenic health effect is not considered serious to cause adverse effects during a person’s lifetime [47]. Moreover, the carcinogenic risk (*CR*_*Cd*_) of the soybean was estimated individually by calculating the cancer probability over a lifetime as the result of exposure to the Cd contaminated soybean. The carcinogenic risk (*CR*_*Cd*_) of Cd in soybean can be evaluated by Eq (3) [43]:

$$CR_{Cd}=ADD_{Cd,ing}\times SF_{Cd,ing}$$ (3)

Where *SF*_*Cd*,*ing*_, is the carcinogenic slope factor for *Cd* (6.1 (kg·day)/mg) via dietary ingestion [45, 46]. Generally, *CR*_*Cd*_ > 1 × 10 ^−04^ is considered to represent a significant cancer risk; 1 × 10 ^−06^ < *CR*_*Cd*_ < 1 × 10 ^−04^ is considered acceptable or tolerable risk; and *CR*_*i*_ or *TCR* < 1 × 10 ^−06^ is negligible risk [45, 48].

#### 2.3.2. Health risk assessment based on the probability distribution

If the HQ and CR values of Cd from soybean suppressed the acceptable range, health risk simulation with the Monte Carlo model was utilized to predict the probability of hazard quotient or carcinogenic risk in a population through numerical modeling and simulation sampling. The advantage of this method is that it can predict the characteristics of population samples with a limited number of samples through the uncertainty factors in the health risk assessment model. This helps objectively evaluate the uncertainty of the health risks, which are consistent with the uncertainty essence of the health risk occurrence. Further, Monte Carlo simulations could overcome the uncertainty of health risk assessment. In this study, we conducted the Monte Carlo simulation to assess the health risks of Cd in soybeans using Crystal Ball, an application in Microsoft Excel. The Monte Carlo simulation was performed using input data in Microsoft Excel spreadsheets [47]. This study defined the concentration of Cd in soybeans in the Eq (1) as the model hypothesis and the hazard quotient or carcinogenic risk as the predictor variable. In the health risk assessment process, the Monte Carlo simulation randomly extracted data with 10000 iterations from the defined assumptions (i.e., the Cd concentration in soybean), and the obtained 10,000 groups of values are used as the input parameters of Eqs (1), (2) or (3), and these results form a new probability distribution function defining carcinogenic risk.

### 2.4. Statistical analysis

The bioconcentration factor (BCF = C _soybean_ / C _soil_) was used to evaluate the Cd accumulation in soybeans from the surrounding soil environment, representing the ratio of soybean Cd to soil Cd [27]. Descriptive statistics of Cd concentrations in soil and soybean samples (such as the mean, maximum, minimum, and standard deviation), multiple linear regression analysis, and neural network models were implemented in the Originpro software (2022 version). The one-way ANOVA with the LSD test was performed to determine the significant difference at the 0.05 level; the Pearson correlation was used to identify various variables correlations. The asterisk indicates significant differences, correlations, and linear regression equations. Crystal Ball software was used to forecast the probability distributions of the HQ or CR values of soybean Cd.

## 3. Results and discussion

### 3.1. Cd accumulation in soil and soybean

**Fig 1:**
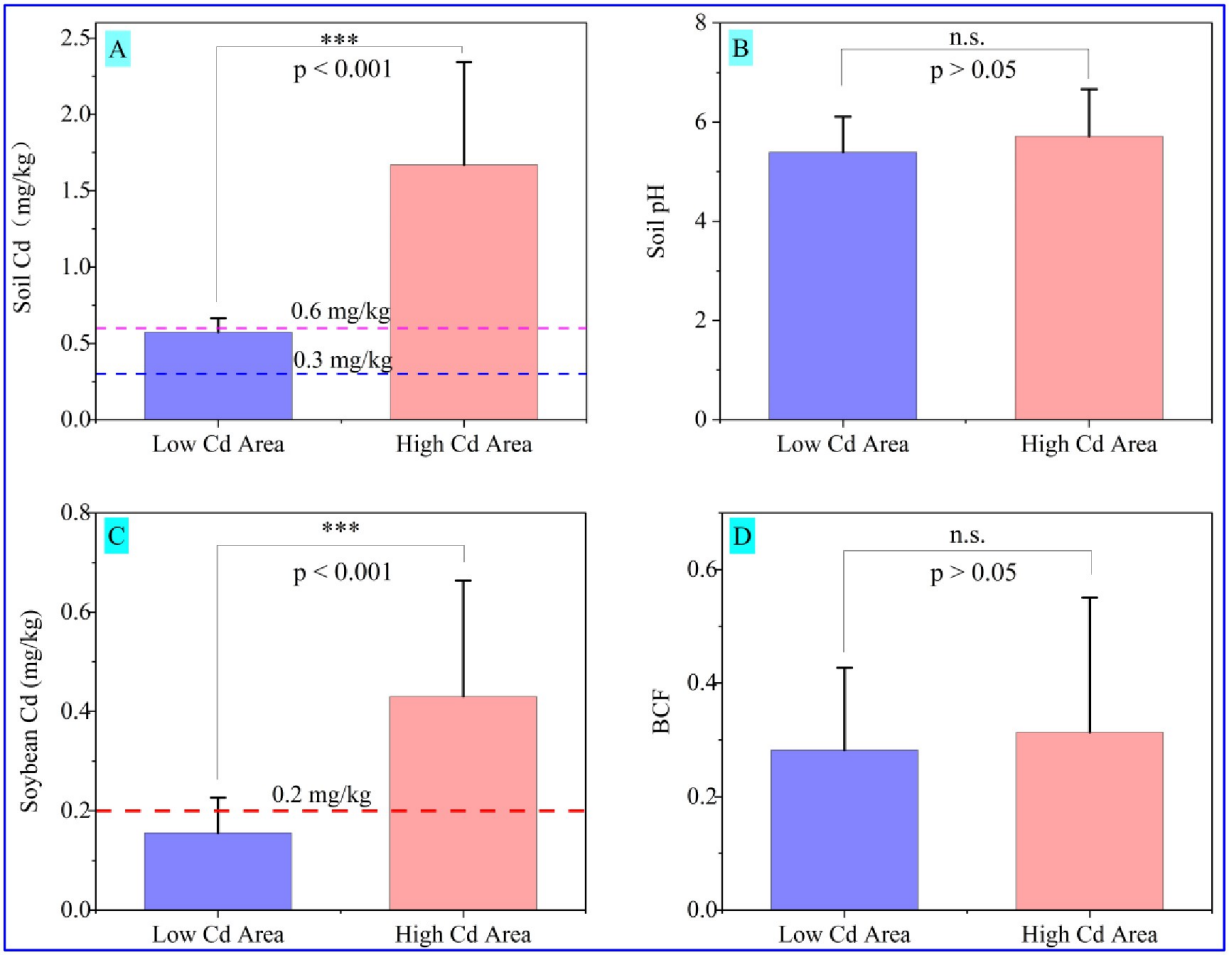
Differences in soil Cd (A), pH (B), soybean Cd (C), and enrichment coefficient (D) in the low- and high-Cd areas. ***above the error bars represent significant differences at the 0.001 level using the LSD test; and n.s. represent no significant differences at the 0.05 level. The blue and pink dashed lines represent the screening values of Cd in agricultural soils at pH ≤ 5.5 and >7.5 in GB15618-2018, China. The red dashed lines represent the standard limit for Cd in soybeans of 0.2 mg/kg as put forth by GB 2762–2017, China.

As shown in Fig 1A, Cd concentrations in the low- and high-Cd areas are 0.639 ± 0.141 mg/kg and 1.726 ± 0.610 mg/kg, respectively. Additionally, the average Cd concentrations in soils around the low- and high-Cd areas were more than one-fold and five-fold of the safety value (0.3 mg/kg obtained from GB15618-2018, China), respectively [49]. The soil Cd concentration in the high-Cd area is significantly higher than in the low-Cd area (*P* < 0.05). Mean Cd concentrations of soil samples in low- and high-Cd areas surpassed the corresponding screening value recommended by the Soil Environmental Quality (GB15618-2018, China) [49], and the proportion of samples exceeding the limit accounted for 100% in Table 1. According to our field investigation results, the areas are located in the karst area in Guizhou province, where groundwater is buried profoundly and is usually not used as irrigation water. The irrigation water in both areas mainly comes from rainfall, without the impact of groundwater contaminated by industrial pollution. From the variation in soil Cd of standard deviation, we can speculate that the spatial distribution of Cd in both areas is an apparent heterogeneity. In general, atmospheric dry and wet deposition contributes significantly to soil Cd, especially in agricultural soils around industrial areas [27, 50]. However, In the small-scale study areas, where climatic conditions and air quality are homogeneous, the contribution of dry and wet deposition to soil Cd accumulation should generally be uniform. If the dry and wet deposition is dominant to the soil Cd accumulation, then there should be no high heterogeneity in both areas with a small scale. Therefore, It can be speculated that the main contributor to the high Cd concentration in soils in both areas should not be dry and wet sedimentation. Previous studies have shown that typical geological anomalies cause high Cd content in soils in Guizhou Province in China, derived from sedimentary parent materials (especially Cd-rich black limestone) [51]. The high-Cd contents without human sources verified that both regions are located in the high geological background area in Guizhou province (Cd background reference values of 0.66 mg/kg) [35, 51]. These results indicated a noticeable difference in the characteristics of soil Cd accumulation in both regions. As shown in Fig 1B, the soil pH in the high-Cd area (pH = 5.71 ± 0.95) is not significantly higher than that in the low-Cd area (pH = 5.39 ± 0.72) (*P* > 0.05). The ranges of soil pH in low- and high-Cd areas are 4.66–7.31 and 4.60–8.19, respectively, indicating that soils in both regions are acidic to weakly alkaline. To our knowledge, soils in the study area belong to the calcareous soil enriched with calcium carbonate minerals, which have a good buffer effect on soil acidity. The difference in soil acidification degree in this study might be ascribed to the following reasons: 1) the application of nitrogen fertilizer; 2) the loss of calcium and magnesium ions and the enrichment of iron and aluminum ions by weathering and leaching; 3) differences in organic acids secreted by crop roots; and 4) sulfide-rich black rock series producing acid through weathering [52, 53].

**Table 1:** Concentrations of Cd in soil and soybean samples (N = 36).

Classification		Mean (mg/kg)	SD (mg/kg)	Min (mg/kg)	Max (mg/kg)	Limit value (mg/kg)	*P* (%)
Soil Cd	Low-Cd Area	0.573	0.092	0.411	0.732	0.3 ^a^ /0.6 ^a^	100.00
	High-Cd Area	1.688	0.686	0.798	3.12	0.3 ^a^ /0.6 ^a^	100.00
Soybean Cd	Low-Cd Area	0.156	0.071	0.036	0.242	0.20 ^b^	61.11
	High-Cd Area	0.430	0.234	0.123	0.839	0.20 ^b^	83.33

Note: P (%) represents the percentage of points exceeding the standard value; SD represents the standard deviation.

^a^. Soil environmental quality in China: Risk control standard for soil contamination of agricultural land (GB15618-2018).

^b^. National standards for food safety in China: Limits for contaminants in food (GB 2762–2017).

The differences in the Cd content of soybean and its enrichment capacity in both regions are plotted in Fig 1C and 1D. The Cd content of soybean in the high-Cd area (0.430 ± 0.234 mg/kg) was significantly higher than that in the low-Cd area (0.156 ± 0.071 mg/kg) in Fig 1C (*P* < 0.05). The percentages of soybean samples whose Cd exceeded the limit values of food standards (0.2 mg/kg from the GB 2762–2017, China) in high- and low-Cd areas were 83.33% and 61.11%, respectively (Table 1). These results indicated that the potential risk of dietary intake from Cd-enriched soybeans might exist in areas with a high Cd geological background. Previous studies have found that the Cd content of soybean growing in high Cd contaminated soils (21 mg/kg) up to 0.76 mg/kg, which is much higher than in other countries (such as Argentina, Germany, the United States, and Brazil) [7]. As shown in Fig 1D, the BCFs of soybean for Cd in low- and high-Cd areas were 0.282 ± 0.145 and 0.314 ± 0.237, respectively. Usually, the enrichment capacity of soybean for Cd is higher than other toxic elements (such as Hg, As, Pb, and Cr) [9, 10], suggesting that soybean is liable to absorb soil Cd and accumulate it in soybean grains. Based on these results, we can conclude that soybeans probably experienced Cd contamination in both areas with geological background anomalies. In addition, there was no significant difference in the BCF of soybean for Cd in low- and high-Cd areas (*P* > 0.05), implying that the Cd content in soils was not the only critical factor determining soybean enrichment ability. Previous studies show that plant Cd content is related to the total concentration and bioavailability of soil Cd [54]. Cd bioavailability in soils is often associated with its fractions, including the soluble fraction, Fe/Mn oxyhydroxide fraction, organically bound fraction, and residual fraction [55]. These fractions depend on the soil pH, organic matter, and minerals. To our knowledge, the available Cd in soils is an essential and direct source of Cd absorbed by crops [56]. The available Cd (such as CaCl_2_-extractable, acetic acid-extractable, and water-extractable Cd), which is regulated by the soil pH, is another crucial factor and determines the Cd content of soybean. Weak acid-bound Cd in soils predominates the proportion of total Cd in soils, indicating that Cd in agricultural soils usually poses a high potential ecological risk and is easy to transfer to crops [55, 57, 58].

### 3.2. Responses of soybean Cd accumulation to soil Cd and soil pH

**Fig 2:**
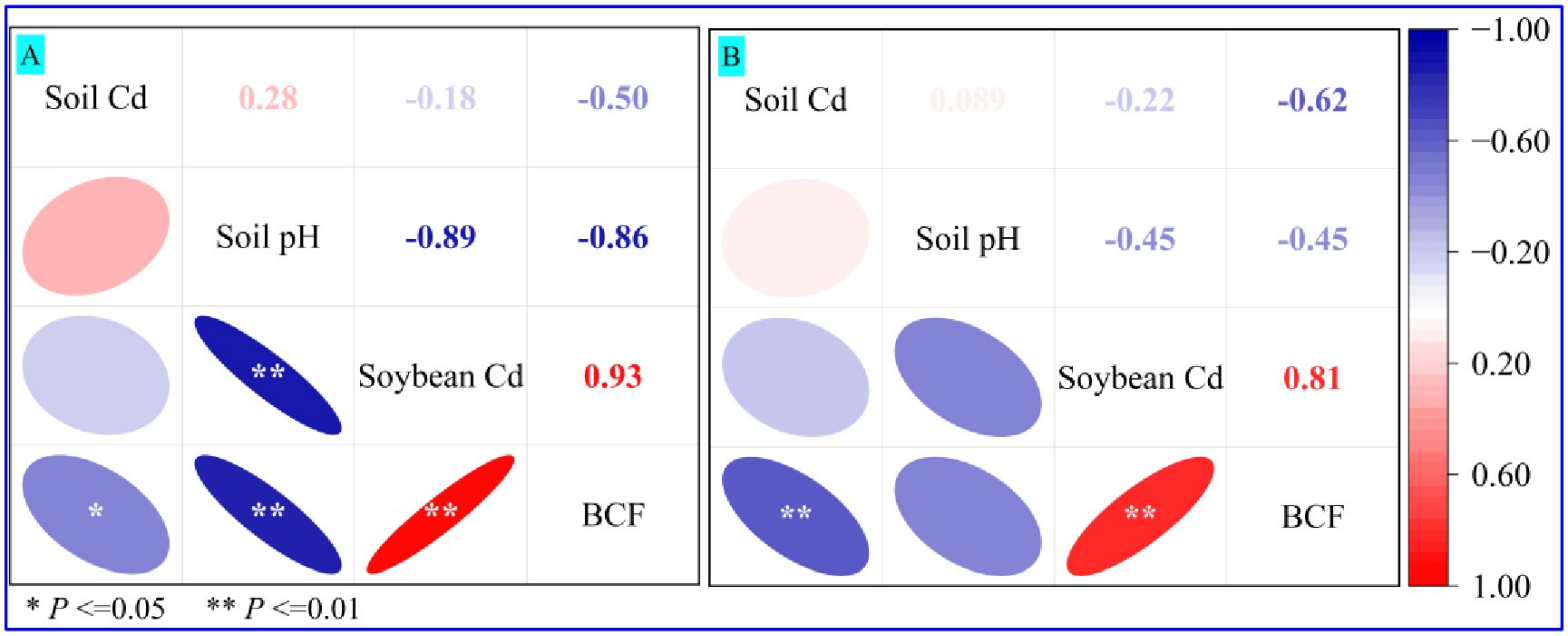
Correlation analysis among soil Cd, soil pH, soybean Cd, and soybean BCF in the low- (A) and high- (B) Cd areas. The number indicates the Pearson correlation coefficient and the color indicates the magnitude of the coefficient. Ellipses represent the dispersions of the samples; * and ** represent the significant correlation at the 0.05 and 0.01 levels, respectively.

The correlation analysis of soil Cd, soil pH, soybean Cd, and BCF are illustrated in Fig 2. In the low-Cd area (Fig 2A), with increases in the soil Cd, BCFs of the soybean significantly decreased (*P* < 0.05, *R* = – 0.50*); with the rise in the soil pH, soybean Cd (*P* < 0.01, *R* = – 0.89**) and soybean BCF (*P* < 0.01, *R* = – 0.86**) also decreased significantly. The Cd concentration and BCF of soybean exhibited a significant positive correlation (*P* < 0.01, *R* = 0.93**). As illustrated in Fig 2B, the soybean BCF presented a significant decrease with the increase in the soil Cd (*P* < 0.01, *R* = – 0.62**) and the reduction of Cd content in soybean (*P* < 0.01, *R* = 0.81**) in the high-Cd area. However, there was no significant relationship between soil Cd and soybean Cd in the low-Cd area (*P* > 0.05, *R* = – 0.18) and high-Cd area (*P* > 0.05, *R* = – 0.22) in Fig 2. When comparing the correlation analysis between soil Cd and soybean Cd in the low- and high-Cd areas, the results showed that the bioaccumulation capacity of soybean gradually decreased with increasing soil Cd concentration and soil pH [10].

**Fig 3:**
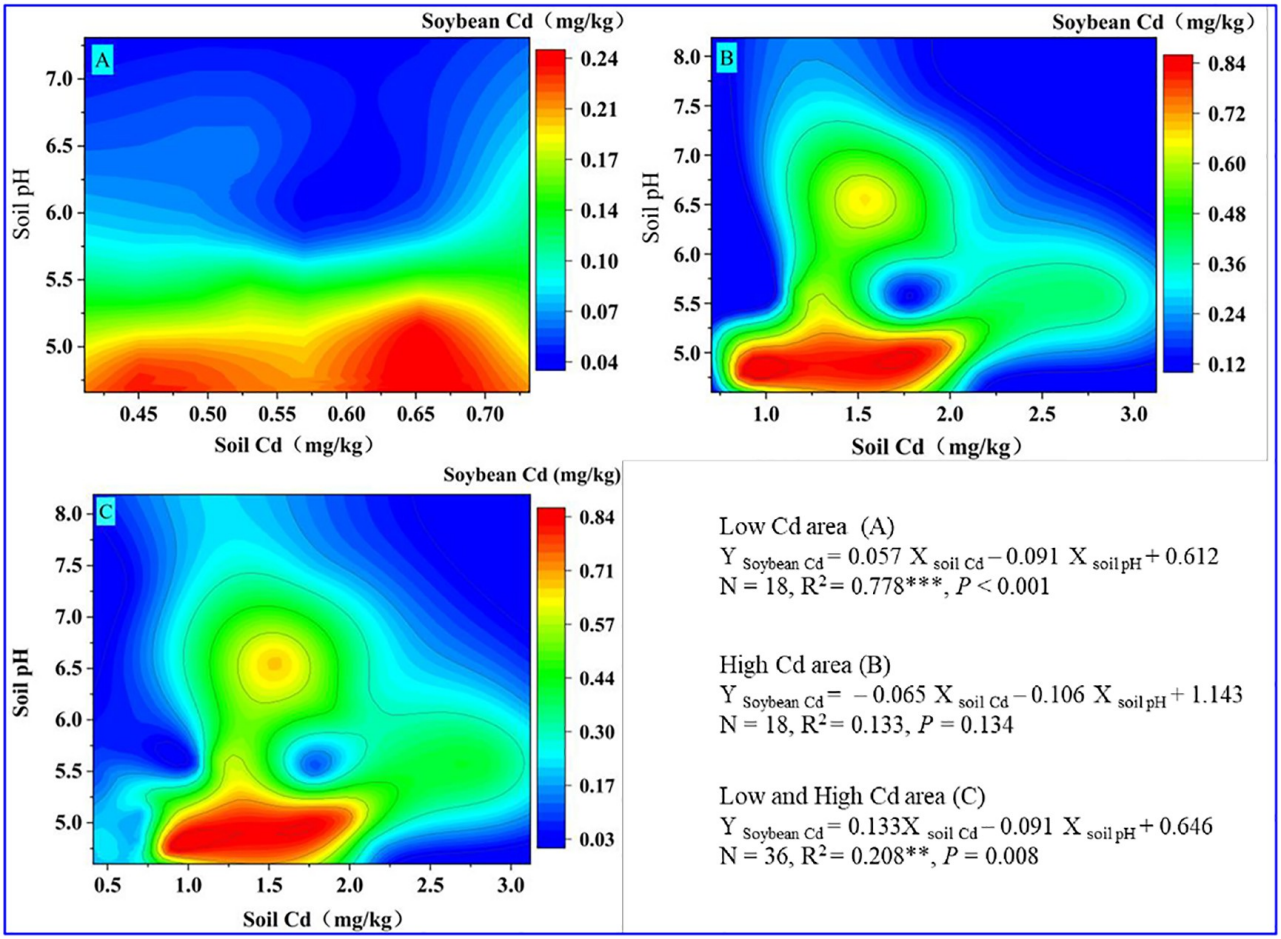
Response of soybean Cd content to soil Cd versus soil pH in low-Cd area (A), high-Cd area (B), and both areas (C), respectively. The color legend indicates the size of the soybean Cd concentration. The equation represents the multiple linear regression results of soybean Cd regarding soil pH and soil Cd. The R^2^ value represents the determination coefficient of the equation, and P < 0.05 indicates that the regression equation has statistical significance at the 0.05 level.

Based on the correlation analysis, we further studied the response of soybean Cd and BCF to soil Cd *versus* soil pH. Fig 3A shows that soybeans with high Cd content mainly gathered in low pH soils (< 5.5) in the low-Cd area. Furthermore, the results of multiple linear regression (Y _Soybean Cd_ = 0.057 X _soil_ Cd− 0.091 X _soil pH_ + 0.612, *R*^2^ = 0.778***, *P* < 0.001) showed that soil pH contributed much more to Cd accumulation in soybean than the soil Cd concentration. As illustrated in Fig 3B, soybean with high Cd content was mainly gathered from soils with low pH soils (pH< 5.5) in the high-Cd area; in addition, the results of multiple linear regression (Y _Soybean Cd_ = – 0.065 X _soil_ Cd− 0.106 X _soil pH_ + 1.143, *R*^2^ = 0.133, *P* = 0.134) showed that soil pH also contributed more to Cd accumulation in soybean than soil Cd concentration. The soybean presented a low Cd accumulation in soils with high pH, proving that the soil pH determined the Cd accumulation of soybeans in both areas. Recent research has also suggested that the mean concentration of Cd in soybeans worldwide is negatively correlated with the soil pH (Y _Soybean Cd_ = – 0.313X _soil pH_ + 0.972, *R*^2^ = 0.650, *P* < 0.001, N = 10) [7], which is consistent with the results of our study (Y _Soybean_ Cd = 0.133 X _soil Cd_− 0.091 X _soil pH_ + 0.646, *R*^2^ = 0.208, *P* = 0.008, N = 36, Fig 3C). These results imply that regulating the soil pH could effectively control the Cd accumulation of soybeans in the high Cd geological background area.

**Fig 4:**
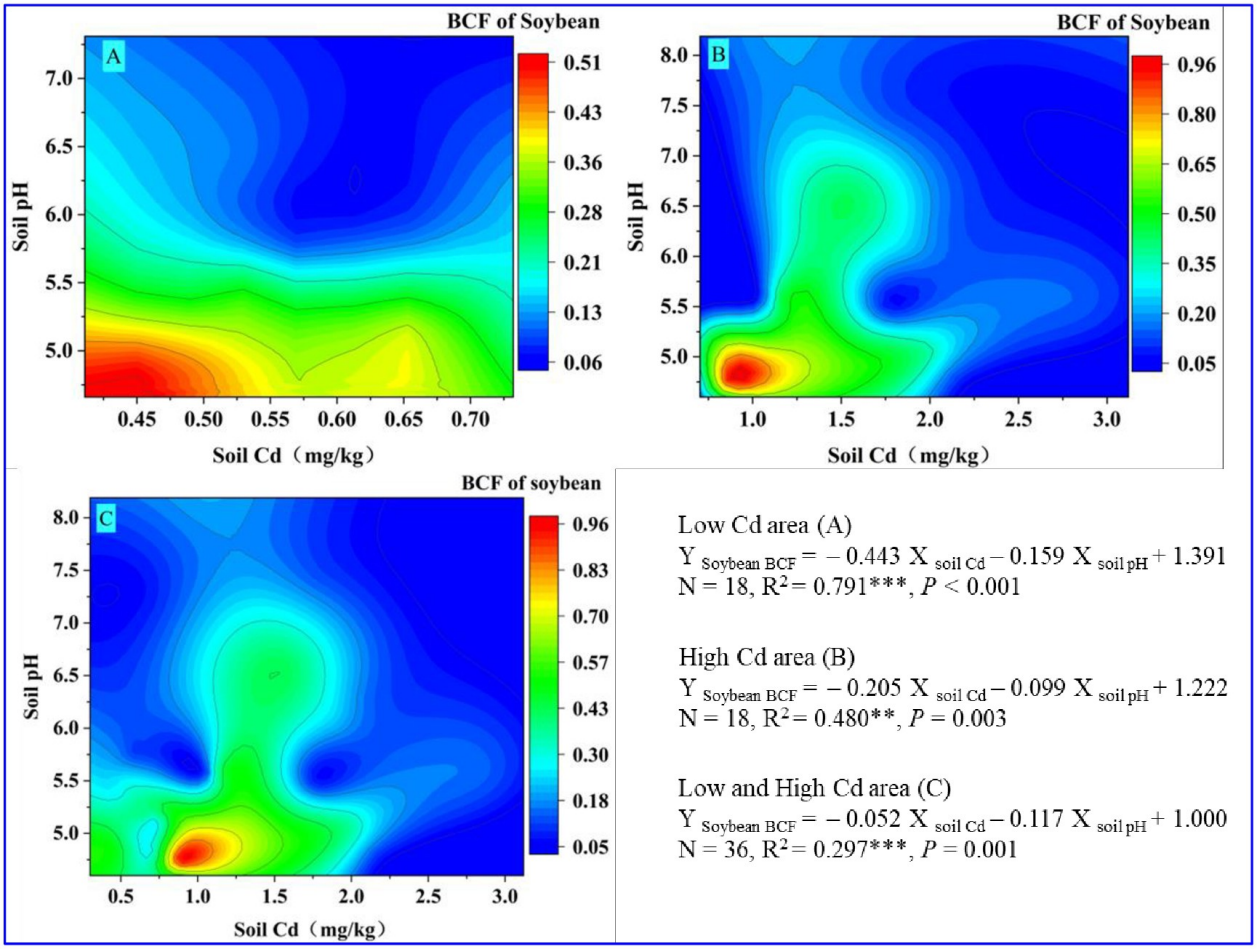
Response of BCF of soybean Cd to soil Cd versus soil pH in the low-Cd area (A), high-Cd area (B), and both areas (C), respectively. The color legend indicates the size of the BCF of soybean Cd. The equation represents the multiple linear regression results of BCF of soybean Cd regarding soil pH and soil Cd, respectively. The R^2^ value represents the determination coefficient of the equation, and P < 0.05 indicates that the regression equation has statistical significance at the 0.05 level.

As shown in Fig 4, the bioaccumulation capacity of soybeans for Cd is affected by soil Cd *versus* soil pH. High BCF is mainly distributed in soils with a low Cd concentration and pH value in the low-Cd area. The multiple linear regression of soybean BCF in the low-Cd area (Y _Soybean BCF_ = – 0.443 X _soil_ Cd− 0.159 X _soil pH_ + 1.391, *R*^2^ = 0.791***, *P <* 0.001, Fig 4A) and high-Cd area (Y _Soybean BCF_ = – 0.205 X _soil_ Cd− 0.099 X _soil pH_ + 1.222, *R*^2^ = 0.480**, *P* = 0.003, Fig 4B) clarified that no matter in low- or high-Cd soils, the effect of soil Cd concentration on BCF of soybean was more evident than soil pH. Compared to the soil pH, the soil Cd concentration had a more significant contribution to the bioaccumulation capacity of soybeans in both areas. The regression coefficient of soil Cd via multiple linear regression analysis demonstrated that the bioaccumulation capacity of Cd in soybean gradually decreased with increasing soil Cd concentration [10], which is consistent with the results of the correlation analysis in Fig 4C. We used the neural network model to further verify the function of soybean Cd with the soil Cd *versus* soil pH. The results suggested that the soil pH and total soil Cd could reasonably predict the Cd accumulation of soybean in S3 Fig (*R* = 0.989, *RMSE* = 0.033).

These results demonstrate that soil acidification and soil Cd accumulation also contribute to Cd uptake and accumulation and are likely the main factors contributing to the high Cd content in soybeans. The correlation analysis and multiple linear regression analysis showed that the Cd content and Cd bioaccumulation capacity of soybeans were determined by soil Cd content and soil acidification degree [59]. When the acid-base reaction of soils changes slightly during the soybean growth, the soil Cd bioavailability could vary significantly. For example, an increase in pH of 1 unit could decrease the Cd concentration of soybean by 1.1 units [7]. The acid-base reaction, which is dominated by proton transfer, controls the Cd bioavailability in cultivated soils via the adsorption competition between H^+^ and Cd^2+^ on the soil matrix and carbonate consumption in calcicolous soils. Usually, soil Cd could be transformed into a low bioavailable fraction of Cd(OH)_2_ in soils with a high pH value [55] or CdCO_3_ in calcareous soils [60]. In addition, the acidification process of agricultural soils is mainly a consequence of atmospheric wet deposition and fertilizer use. Acid rain inverted acidified soil may affect the growth and quality of soybeans [61]. Guizhou is an acid rain-prone area, and it can activate the Cd from weakly acid-bound fraction to plant-available fraction. Therefore, the long-term monitoring of acid-base properties of Cd-contaminated soils should be conducted because of the large amount of available Cd (weak acid-bound Cd) in agricultural soils in areas with high geological background.

### 3.3. Health risk assessment of Cd in soybean

**Fig 5:**
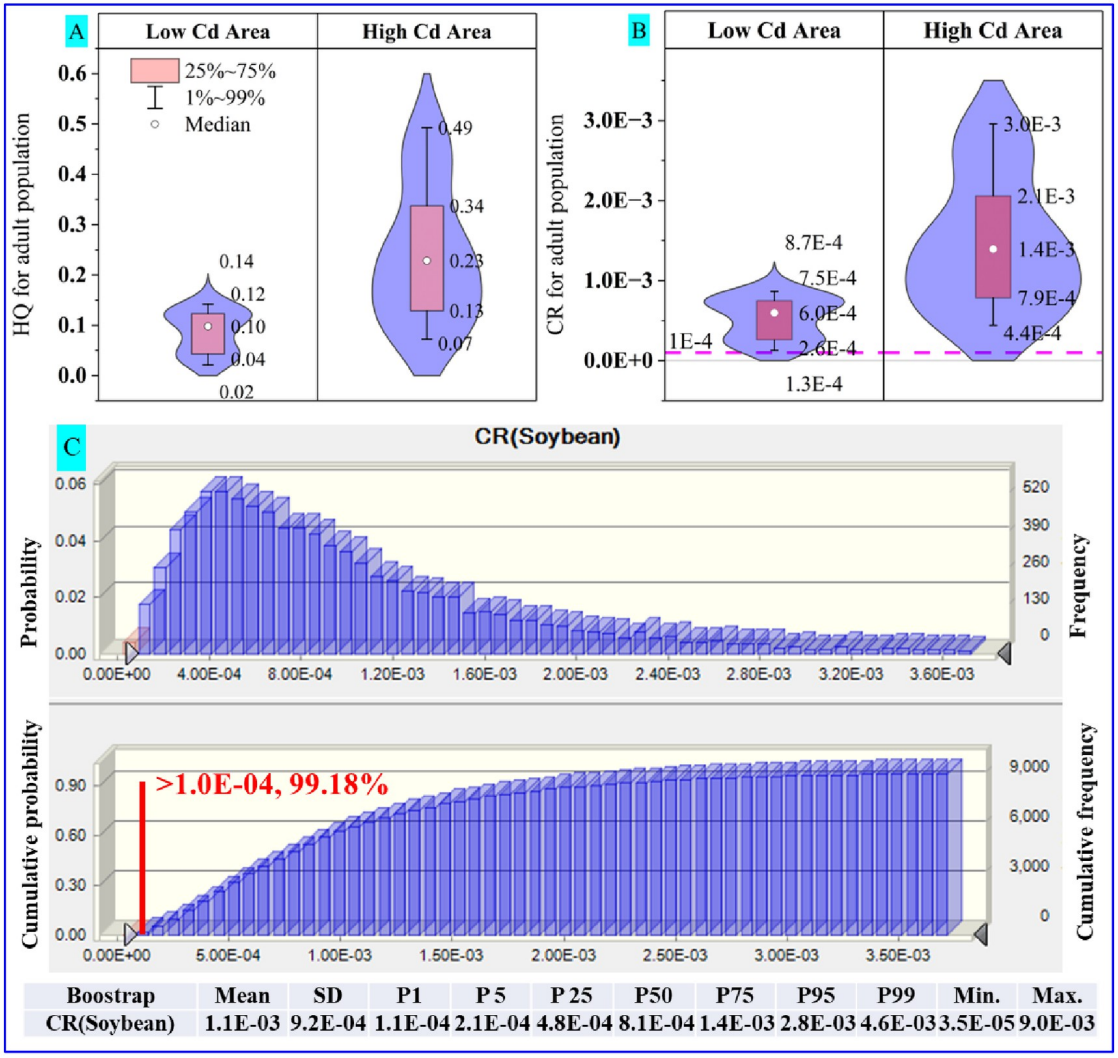
HQ values (A) and CR values (B) of soybean for the adult population and the corresponding probability distribution of CR values in low- and high-Cd areas (C). The pink dotted line represents 1.0E-04. The violin plot, from the bottom to the top, represents one percentile (P1), 25 percentile (P25), 75 percentile (P75), and 99 percentile (P99), respectively. The white circle represents the median. The probability and cumulative probability of the CR value from the ingestion of Cd in soybean (low and high-Cd areas, N = 36) were obtained via Monte Carlo simulation. CR values were evaluated using a Monte Carlo simulation in Crystal Ball with 10000 iterations. The bottom table shows the CR value for the adult population from soybean ingestion in the Monte Carlo Simulation study with a bootstrap of 200 sample capacity and 1000 time tests for each sample.

The non-carcinogenic and carcinogenic risks for adults were determined for the exposure calculation, as plotted in Fig 5. The HQ values of Cd from soybean at 99 percentiles in the low- and high-Cd areas were 0.14 and 0.49, respectively. The adult population who ingested the Cd-contaminated soybean in low- and high-Cd areas did not suffer from adverse non-carcinogenic risk, maintaining an acceptable health risk range (99 percentile HQ value of < 1.0) in Fig 5A. Furthermore, as shown in Fig 5B, the median, P1, P25, P75, and P99 of CR values from soybean ingestion in the low-Cd area were 6.0 × 10 ^−04^, 1.3 × 10 ^−04^, 2.6 × 10 ^−04^, 7.5 × 10 ^−04^, and 8.7 × 10 ^−04^, respectively, and in the high-Cd area were 1.4 × 10 ^−03^, 4.4 × 10 ^−04^, 7.9 × 10 ^−04^, 2.1 × 10 ^−03^, and 3.0 × 10 ^−03^, respectively. Although the mean concentration of Cd in soybeans in the low-Cd area did not exceed the limits of the National Food Safety Standard (GB 2762–2017), they still might induce carcinogenic risks in the residents, and this phenomenon has often been found in other studies as well [10]. CR values based on the total Cd concentration in soybeans suggested that the health risks in low- and high-Cd areas were still much higher than the acceptable range (< 1.0 × 10 ^−04^) [47]. Monte Carlo model results suggested that the CR value from soybean Cd was much higher than the acceptable range, and the corresponding accumulated percentiles of CR value > 1.0 × 10 ^−04^ was 99.18% in Fig 5C. In other words, residents’ exposure to Cd-contaminated soybean consumption might cause an unacceptable carcinogenic risk in both areas with high geological backgrounds.

According to the relationship between the soybean Cd and soil pH in **Section 3.2**, soil acid-base properties should be prioritized when developing remediation strategies for Cd-contaminated soil. Other factors should also be considered, such as the low Cd accumulation of soybean varieties and soil Cd immobilization of the passivation materials [16, 17]. The meta-analysis clarified that CaCO_3_ as a passivator is more efficient in evaluating the soil pH and inhibiting the Cd accumulation in plant shoots than CaO and Ca(OH)_2_ [21]. The increase in soil pH from 5.5 to 6.5 caused by applying 7.5 t/ha CaCO_3_ exhibited excellent efficiency in reducing the Cd accumulation of 70%– 80% [56, 62]. When the soil pH reached 8, the available Cd in soils could transform into astable and low bioavailable fraction of Cd(OH)_2_) [55] or of CdCO_3_ in calcareous soils with a large amount of CaCO_3_ [60].

**Fig 6:**
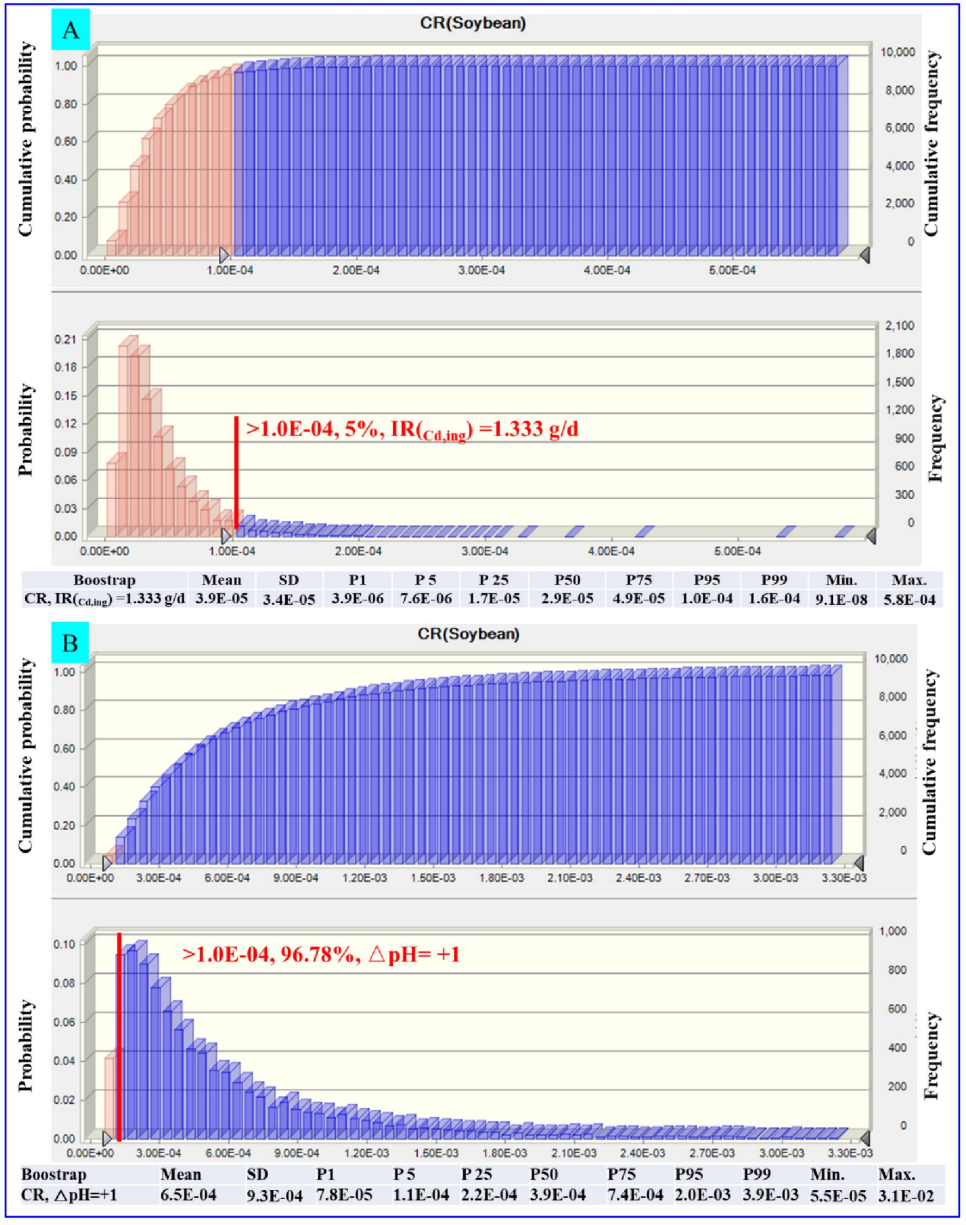
Health risk prediction based on two hypothetical scenarios of the Carlo model simulation. The safe intake dose of soybean was estimated at current contamination concentrations using the Monte Carlo model at the CR value of P95 = 1.0 × 10−^04^ (A). The CR value of Cd in soybean was evaluated by raising the soil pH (ΔpH = +1) (B). CR values were computed using a Monte Carlo simulation in Crystal Ball with a bootstrap of 200 sample capacities and 1000 tests for each sample.

In the current state of soybean contamination, we set up two scenarios for reducing cancer risk (i.e., reducing the intake of soybean and increasing soil pH by 1 unit) to discuss the feasibility of common mitigation measures. Firstly, as shown in Fig 6A, the soybean consumption dose should decrease to 1.333 g/d (CR value of P95 = 1.0 × 10 ^−04^) to keep residents from soybean Cd carcinogenic risks. Such a low recommended intake seems incompatible with the residents’ dietary habits, making it difficult for them to change their dietary structure. Secondly, assuming that soil pH is raised by 1 unit, we predicted whether Cd in soybeans is an acceptable health risk to residents with the combination of the neural network model in S3 Fig and the Monte Carlo model in Fig 6B. According to Fig 6B, the carcinogenic risk of exposure to Cd in soybean (CR value of P95 = 2.0 × 10−^03^) is still unacceptable under a 1 unit increase of soil pH, indicating that the increase in soil pH does not necessarily protect soybean from excessive Cd accumulation and then protect the resident from the health risks associated with Cd-contaminated soybean ingestion. Under the unchanged dietary structure, the combined mitigation strategies are essential to diminish the Cd accumulation in soybeans to achieve acceptable health risks, such as using the passivating agents to adsorb and immobilize the soil Cd and screening low-Cd accumulation soybean varieties.

Therefore, further research should focus on the following aspects: **1)** screening the suitable soybean varieties with the low-Cd accumulation capacity in the high Cd geological background area; **2)** conditioning the acidified soils with the alkaline amendment (e.g., biochar and calcium carbonate) to reduce the solubility of Cd-containing compounds and the Cd bioavailability in acidified or acidic soils; **3)** conducting a detailed survey of Cd accumulation in soybeans and an analysis of its health risks in the high Cd geological background areas nationwide; and **4)** proposing other remediation strategies, such as the adjustment of crop planting structure and a reduction of the residents’ intake of high Cd crops.

## 4. Conclusions

This study discussed the accumulation characteristics of Cd in soybeans and the corresponding health risks for the adult population. The findings indicate that Cd-contaminated soybeans (> 0.2 mg/kg) in low- and high-Cd areas pose an unacceptable carcinogenic risk (CR value > 1.0 × 10 ^− 04^) for the adult population. The accumulation characteristic of Cd in soybeans in both regions could be explained well using the soil pH and soil Cd via multiple linear regression and neural network models. These results imply that regulating the soil pH could effectively reduce the Cd accumulation of soybeans in the high Cd geological background areas. However, our discussion found that the reduced soybean intake and increased soil pH at a reasonable level did not effectively avoid the carcinogenic risk from soybeans Cd to an acceptable range. This requires the exploration of more efficient remediation strategies, such as screening soybean varieties with low-Cd accumulation and other comprehensive remediation strategies. Further, the research result reminds us that directly planting soybeans in soils in high Cd geological background areas is not recommended unless new remediation strategies are combined with low-Cd accumulation soybean varieties.

## Supporting information

S1 Fig Distribution of sampling points. (DOCX)

S2 Fig Flow chart of this study. (DOCX)

S3 Fig The fitted results of soybean Cd variation with soil Cd *versus* soil pH via the neural network model (N = 36). The model included three layers (4, 7, and 4 neurons, respectively), maximum iterations of 10000, a learning rate of 0.01, and the activation function of the Sigmoid Function, f (x) = 1 / (1 + exp (-x)).
(DOCX)
