# Supplementary figures and images for: Response of soybean Cd to soil Cd and pH and its associated health risk in a high geological background area in Guizhou Province, Southwest China
Source: PLoS One. 2024 Oct 22;19(10):e0312301. doi: 10.1371/journal.pone.0312301 (PMC11495549; doi:10.1371/journal.pone.0312301)

**Figure S1.** **Distribution of sampling points.**

**
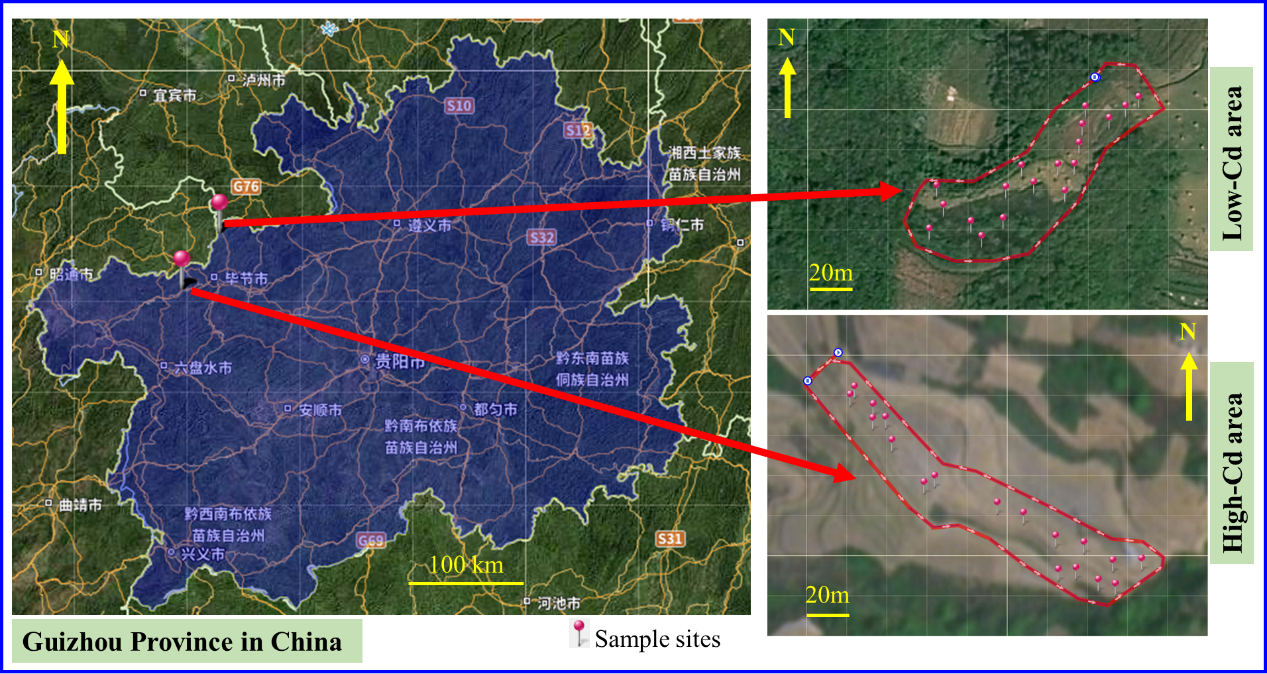
**

Supplement: S1 Fig — (DOCX) [file pone.0312301.s001.docx]

**Figure S2 Flow chart of this study**


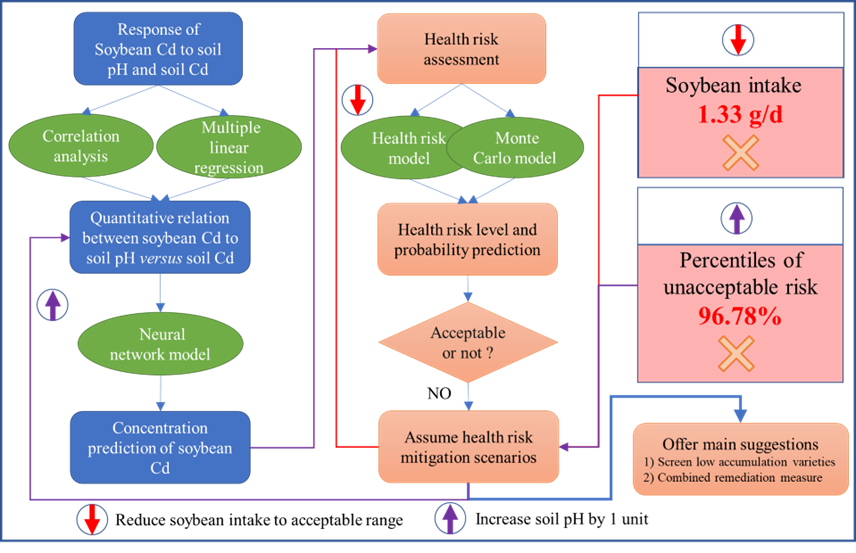

Supplement: S2 Fig — (DOCX) [file pone.0312301.s002.docx]
