# Supplementary material for: Response of soybean Cd to soil Cd and pH and its associated health risk in a high geological background area in Guizhou Province, Southwest China
Source: PLoS One. 2024 Oct 22;19(10):e0312301. doi: 10.1371/journal.pone.0312301 (PMC11495549; doi:10.1371/journal.pone.0312301)

**Figure S3.** **The fitted results of soybean Cd variation with soil Cd *versus* soil pH via the Neural network model (N = 36)**. The model included three layers (4, 7 and 4 neurons, respectively), maximum iterations of 10000, a learning rate of 0.01, and the activation function of the Sigmoid Function, f (x) = 1 / (1 + exp (-x)).


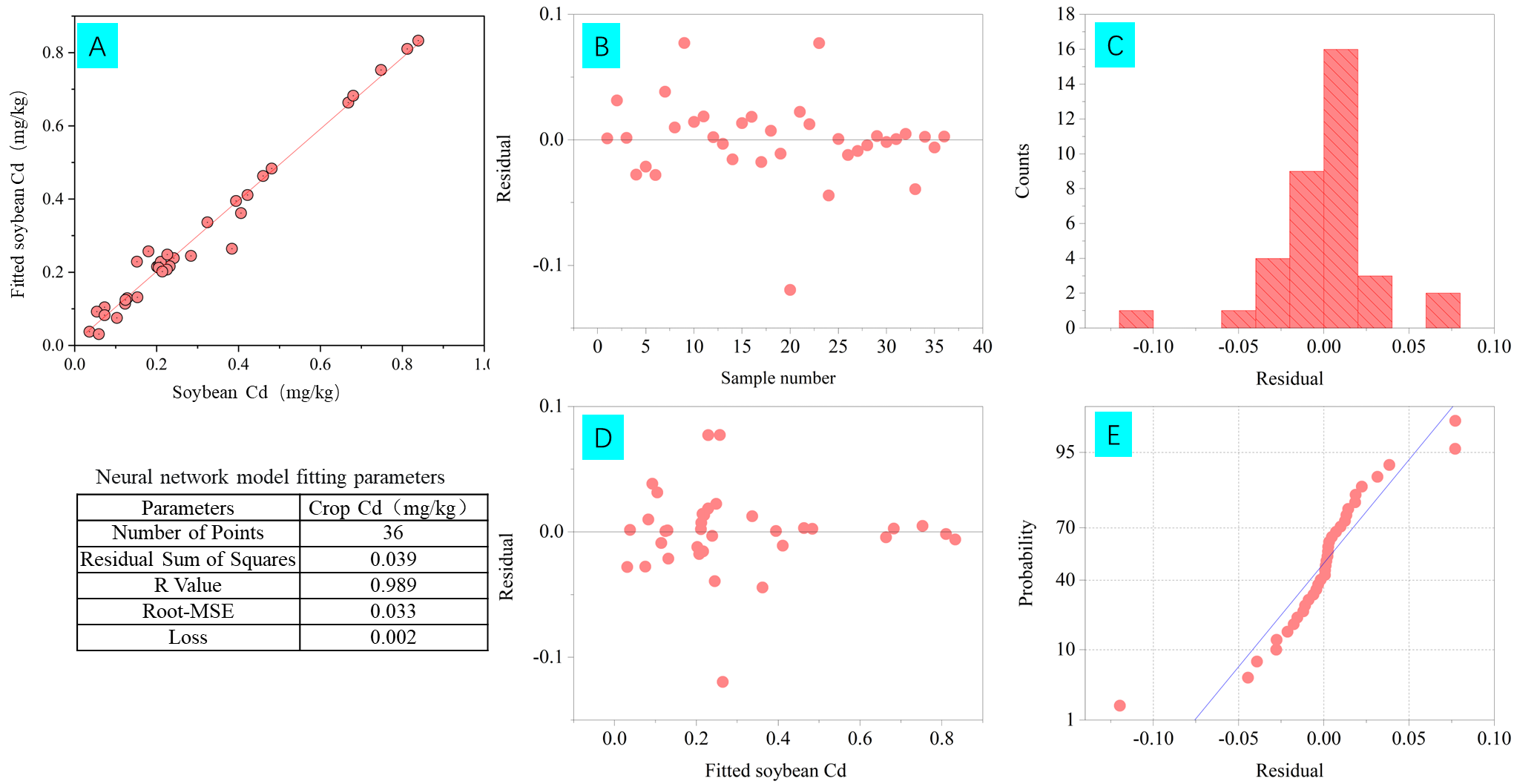

Supplement: S3 Fig — The model included three layers (4, 7, and 4 neurons, respectively), maximum iterations of 10000, a learning rate of 0.01, and the activation function of the Sigmoid Function, f (x) = 1 / (1 + exp (-x)). (DOCX) [file pone.0312301.s003.docx]
